# The Practitioner

**Published:** 1880-02

**Authors:** 


THE PRACTITIONER.—Our confrère of the St. Louis *Clinical Record* evidently thinks there *is something* in a name, and handles us *sans cérémonie* for adopting ours. In christening *our* bantling we thought we could adopt any name we might elect in this "land of the free and home of the brave," that should not be offensive to ears polite, nor encroach upon or appropriate the goods of our neighbors! That neither has been done is quite evident from the list he furnishes of journals bearing in whole or part our cognomen. These have not complained, so far as we are advised, of the name we bear. But the prefix of the *definite* article, and the *place* of publication, ought to individualize us sufficiently for all useful or practical purposes, and probably as little ambiguousness will be experienced in forwarding or referring to us as in the case of any other journal. Certainly no more need occur in either of these respects in regard to us, than to several others that have had no more "originality in naming *their* offspring."

But how about the "Record," brother *Clinical* Record, and *its* "originality?" There are *several* John Smiths in Baltimore, none of whom claim, so far as we know, to be the "old original" John. And we would not be at all surprised if a few of this queer old name could be found in the St. Louis City Directory, also. Yet no complaints are heard, if made, on the advent of additional John Smiths in any portion of the country. But we suppose that in names, as in some other things, we should say, *de gustibus*, etc.

